# Automated diagnosis of temporal lobe epilepsy in the absence of interictal spikes

**DOI:** 10.1016/j.nicl.2017.09.021

**Published:** 2017-09-28

**Authors:** Thibault Verhoeven, Ana Coito, Gijs Plomp, Aljoscha Thomschewski, Francesca Pittau, Eugen Trinka, Roland Wiest, Karl Schaller, Christoph Michel, Margitta Seeck, Joni Dambre, Serge Vulliemoz, Pieter van Mierlo

**Affiliations:** a Department of Electronics and Information Systems, Ghent University, Ghent, Belgium; b Functional Brain Mapping Lab, Department of Basic Neuroscience, University of Geneva, Geneva, Switzerland; c Perceptual Networks Group, Department of Psychology, University of Fribourg, Fribourg, Switzerland; d Department of Neurology, Paracelsus Medical University and Center for Cognitive Neuroscience, Salzburg, Austria; e Institute for Diagnostic and Interventional Neuroradiology, University of Bern, Bern, Switzerland; f Neurosurgery Clinic, University Hospital Geneva, Geneva, Switzerland; g Epilepsy Unit, Neurology Clinic, University Hospital of Geneva, Geneva, Switzerland

**Keywords:** Temporal lobe epilepsy, Diagnosis, Lateralization, EEG, Machine learning

## Abstract

**Objective:**

To diagnose and lateralise temporal lobe epilepsy (TLE) by building a classification system that uses directed functional connectivity patterns estimated during EEG periods without visible pathological activity.

**Methods:**

Resting-state high-density EEG recording data from 20 left TLE patients, 20 right TLE patients and 35 healthy controls was used. Epochs without interictal spikes were selected. The cortical source activity was obtained for 82 regions of interest and whole-brain directed functional connectivity was estimated in the theta, alpha and beta frequency bands. These connectivity values were then used to build a classification system based on two two-class Random Forests classifiers: TLE vs healthy controls and left vs right TLE. Feature selection and classifier training were done in a leave-one-out procedure to compute the mean classification accuracy.

**Results:**

The diagnosis and lateralization classifiers achieved a high accuracy (90.7% and 90.0% respectively), sensitivity (95.0% and 90.0% respectively) and specificity (85.7% and 90.0% respectively). The most important features for diagnosis were the outflows from left and right medial temporal lobe, and for lateralization the right anterior cingulate cortex. The interaction between features was important to achieve correct classification.

**Significance:**

This is the first study to automatically diagnose and lateralise TLE based on EEG. The high accuracy achieved demonstrates the potential of directed functional connectivity estimated from EEG periods without visible pathological activity for helping in the diagnosis and lateralization of TLE.

## Highlights

• Both classifiers for TLE diagnosis and lateralization achieved high accuracy (90%).
• Outflow from left and right hippocampus was the most important for diagnosis.
• Outflow from the right ACC was the most important for lateralization.
• The interaction between features is important for a correct classification.

## 1 Introduction

Mesial temporal lobe epilepsy (TLE) is the most common type of pharmaco-resistant epilepsy in adults. In order to estimate the localisation of the epileptogenic zone, Electroencephalography (EEG) is recorded to identify pathological activity such as seizures or Interictal Epileptiform Discharges (IEDs). However, in some patients, these are infrequent or completely absent in the EEG recording.

Epilepsy is increasingly recognized as a network disease (Laufs, 2012) and measures of functional relationships between activities of different brain regions could help better understand epileptic networks. Directed functional connectivity estimates the directionality of the functional connections between different brain regions. Several studies have shown that directed functional connectivity measures based on intracranial EEG can help to identify the irritative zone and the seizure onset zone (van Mierlo et al., 2013, Wilke et al., 2009, van Mierlo et al., 2014). Furthermore, directed functional connectivity applied to brain sources estimated from high-density scalp EEG revealed interictal network patterns concordant with cognitive deficits in TLE (Coito et al., 2015) and significant connectivity differences in TLE compared to healthy controls in the absence of interictal spikes (Coito et al., 2016).

Machine learning algorithms have been used for automatic detection and localization of the epileptogenic zone in TLE using a multitude of imaging modalities (Focke et al., 2012, Kamiya et al., 2016, Cantor-Rivera et al., 2015, Chiang et al., 2015, Yang et al., 2015, Kerr et al., 2013). However, to the best of our knowledge, no study has attempted to automatically diagnose and lateralise TLE using scalp EEG.

Here, we used EEG-based directed functional connectivity values to build a diagnostic and lateralization classification system for TLE in the absence of visible epileptic activity. Moreover, we compared our results with previous classification studies using other imaging modalities.

## 2 Materials and methods

### 2.1 Subjects

Twenty LTLE patients, 20 RTLE patients and 35 healthy subjects were included. Patients were retrospectively selected from the high-density EEG database of the University Hospital of Geneva, University Hospital of Bern and Paracelsus Medical University in Salzburg according to the following inclusion criteria: drug-resistant TLE, unilateral anteromedial localization of the epileptogenic zone confirmed by good surgical outcome (Engel's class I or II, after at least 12 months post-operative follow-up), intracranial EEG or concordant presurgical evaluation methods and the existence of at least a 10–15 min resting-state eyes-closed high-density EEG recording (96–256 channels). All patients had interictal activity on long-term EEG concordant with the diagnosis of unilateral TLE. Most of them had extensive presurgical evaluation including ictal video-EEG, PET, SPECT and electric source imaging. The patients' dataset used in this study was the same as reported in our previous work (Coito et al., 2016). The clinical details can be found in the Supplementary material of the present manuscript.

### 2.2 Standard protocol approvals, registrations, and patient consents

All patients were evaluated in the epilepsy units of Geneva University Hospital, Switzerland, Bern University Hospital, Switzerland, and Paracelsus Medical University in Salzburg, Austria. The three local ethics committees approved this study. Written informed consent was obtained from all participants in the study.

### 2.3 EEG, electrical source imaging and directed functional connectivity

Subjects underwent a resting-state eyes-closed recording during presurgical evaluation. The sampling frequency of the recorded EEG ranged between 250 and 1000 Hz. All signals were filtered offline between 1 and 100 Hz and then downsampled to 250 Hz. Sixty epochs of 1 s, free of artefacts and IEDs, during wakefulness were selected per subject. The activity of brain sources during the selected EEG epochs was obtained using Electrical Source Imaging (ESI): an individual head model and a linear distributed inverse solution with biophysical constraints were used (Grave de Peralta Menendez et al., 2004). The grey matter was parcelled in 82 Regions Of Interest (ROI) and the solution point closest to the centroid of each ROI was considered as representative of the source activity in this ROI. This procedure resulted in 82 time-series representing the activity of each individual ROI during the 60 selected epochs.

For each subject and epoch, directed functional connectivity between the 82 source ROIs was estimated using the weighted Partial Directed Coherence (wPDC) (Baccala and Sameshima, 2001, Astolfi et al., 2006, Plomp et al., 2014), and the mean wPDC across epochs was taken.

For each subject, we obtained a 3D connectivity matrix (82 regions × 82 regions × frequency), which represented the outflow from one region to another for each frequency. For further analysis, we reduced the connectivity matrix to 3 frequency bands: theta (4–8 Hz), alpha (8–12 Hz) and beta (12–30 Hz), by calculating the mean connectivity value in each band.

The detailed procedures for EEG recording, electrical source imaging and directed functional connectivity have been described in (Coito et al., 2016) and are also included in the Supplementary material of this manuscript.

### 2.4 Classification

#### 2.4.1 Feature selection

The calculation of the connectivity between every pair of regions in the three frequency bands resulted in 20.172 features for each individual. An optimal subset of these features was selected to avoid creating false decision rules when training the classifier on the example data. As an example, consider the case where a certain connection is slightly stronger for RTLE compared to LTLE patients in the majority of our patients, but not for the whole population of TLE patients. A classifier taking this contingency as a general rule for lateralization can perform poorly on new subjects. This issue of overfitting to example data becomes more likely with decreasing number of subjects and increasing number of feature values per subject (Mwangi et al., 2014, Guyon and Elisseeff, 2003). To avoid overfitting, we allowed a maximum of one feature per ten subjects, resulting in a maximum of 7 features for diagnosis and 4 features for lateralization.

First, the 82 regions were reduced to a set of 14 regions that showed differences between groups in our previous study (Coito et al., 2016) and are known to be involved in TLE: left and right Hippocampus (Hipp), Amygdala (Amyg), Parahippocampus (PHipp), Anterior Cingulate Cortex (ACC), Posterior Cingulate Cortex (PCC), Olfactory cortex (Olf) and Medial Temporal Pole (TPMid). This left us with 588 features that were used to build the first RF classifier. The importance of each feature *f* in this classifier was calculated as the decrease in classification performance when the values of *f* are randomly permuted. As random permutation breaks the link between the feature *f* and the class labels, this permutation importance reflects how much classification power is lost when this feature is taken out of the design of the system. Following the feature selection method by Genuer et al. (2010), features with a non-significant importance were considered irrelevant and thus removed from the set.

Further reduction was obtained by removing redundant information. For that purpose Genuer et al. (2010) selects the minimal subset of features that contains the maximum amount of discriminant information. The method considers the interaction between features during this selection, which is important as the relevance of an outflow may depend on which other outflows were considered as features. For interpretation of the feature selection result, we calculated the actual interaction effect of a feature *f*_*1*_ on another feature *f*_*2*_ as the change in permutation importance of *f*_*2*_ when *f*_*1*_ is removed from the design (again by permuting its values). A negative interaction (a decrease in importance) indicates that the discriminative information in *f*_*2*_ is only relevant when *f*_*1*_ is included in the design. Higher order interactions (e.g. between three features) can also have an impact. However, with increasing order, more data is required to obtain a reasonably accurate measure of interaction. The first order interaction is given here to illustrate the impact of feature interaction in general.

#### 2.4.2 Random Forests

Random Forest (RF) (Breiman, 2001) is a machine learning technique in which an ensemble of elementary classifiers is trained and its outputs aggregated to classify a new input sample. In RF, the ensemble is composed of many classification and regression trees (Loh, 2011), each trained on a different bootstrap subset of the available samples. When a new input is to be classified, each tree in the ensemble makes the classification and the sample is assigned to the class that was chosen by the majority of the trees.

The scikit-learn library (http://scikit-learn.org/stable/) was used to implement a Balanced RF classifier. This classifier differs from standard RF in the way that subsets containing an equal number of subjects from both classes are used to train the decision trees. Every forest contained 1000 trees. The size of the random set of features from which splits were chosen was log_2_(M), where M is the total amount of features per subject. All performance metrics reported in this work were calculated in a Leave-One-Out Cross Validation (LOOCV). In this procedure, each subject is left out of the dataset once, while the others are used for feature selection and classifier training. The classifier system was then tested on the left out subject. In this way, the evaluation illustrates the average performance on a new subject, unseen by the system.

The system built in this work had three output classes: healthy subjects, LTLE and RTLE. Building a three-class classifier with RF is possible but far more complex than building multiple two-class classifiers and combining their results. Moreover, the natural clinical process requires a system in which the subject is first diagnosed with TLE and then, if applicable, the TLE is lateralised. Therefore, we built two separate classifiers, one for diagnosis (TLE vs. healthy subjects) and one for lateralization (LTLE vs. RTLE). Feature selection was done for each classifier individually. The two classifiers were sequentially applied in the final system.

## 3 Results

### 3.1 Classification

The diagnosis classifier achieved an accuracy of 90.7%, sensitivity of 95%, specificity of 85.7% and area under the curve (AUC) of 0.89 (Table 1). For lateralization, the AUC was 0.911 and all other performance measures 90% (Table 1).

**Table 1:** Performance of the diagnosis and lateralization classifiers.

Performance measure	Diagnosis	Lateralization
Accuracy (%)	90.7	90.0
Sensitivity (%)	95.0	90.0
Specificity (%)	85.7	90.0
Positive predictive val. (%)	88.4	90.0
Negative predictive val. (%)	93.8	90.0
AUC	0.890	0.911

Putting the two classifiers in sequence, Table 2 shows the confusion matrix of this three-class classifier system in LOOCV. It shows how the subjects from a certain class were assigned to the three classes by our system. The overall accuracy of the system is 85.3%.

**Table 2:** Confusion matrix for the three-classifier system.

Actual	Predicted		
	LTLE	RTLE	Controls
LTLE	16	2	2
RTLE	2	18	0
Controls	0	5	30

### 3.2 Feature selection

The selected set of features slightly differed between LOOCV iterations due to the intrinsic randomness of the procedure and the RF technique. Table 3 shows the most frequently occurring subset of features, ranked according to their average importance value. For each feature, the p-value of a nonparametric Mann-Whitney-Wilcoxon test is given, testing the null hypothesis that values are equally distributed in the two competing classes. The feature with highest importance for diagnosis was the outflow from the right to the left hippocampus. For lateralization, the outflow from the right ACC to the right hippocampus had the highest importance.

**Table 3:** Feature selection result - selected features for diagnosis and lateralization, sorted from the most to the least important for classification.

Diagnosis			Lateralization		
Feature	Importance (·10^− 2^)	p-Value	Feature	Importance (·10^− 2^)	p-Value
θ Hipp-R → Hipp-L	5.29	0.276	⍺ ACC-R → Hipp-R	9.28	0.068
⍺ Hipp-L → ACC-R	5.23	0.004	θ Hipp-R → Hipp-L	7.58	0.394
β PCC-L → Amyg-R	5.07	0.005	θ TPMid-R → Amyg-R	7.08	0.091
⍺ Hipp-L → TPMid-R	2.52	0.006			
θ Hipp-R → Amyg-R	2.37	0.326			
β ACC-R → TPMid-L	1.33	0.012			

**Fig. 1:**
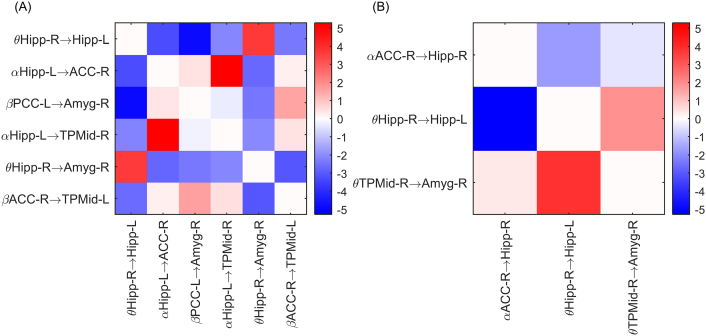
Feature interaction effect - Size of the interaction effect between features for diagnosis (A) and lateralization (B). The color on the intersection of row *f*_*r*_ and column *f*_*c*_ indicates the interaction effect of feature *f*_*r*_ on *f*_*c*_ measured as the drop in feature importance of *f*_*c*_ when *f*_*r*_ is left out of the design. Blue means a negative interaction, the discriminative information in *f*_*c*_ is less relevant when *f*_*r*_ is excluded from the design. Red means a positive interaction, the discriminative information in *f*_*c*_ becomes more relevant when *f*_*r*_ is excluded from the design. (For interpretation of the references to color in this figure legend, the reader is referred to the web version of this article.)

Fig. 1 shows the interaction between the selected features. For diagnosis, the interaction between the outflow from the right to the left hippocampus and the outflow from the right hippocampus to the right amygdala were the most important. For lateralization, the interaction between the outflow from the right to the left hippocampus and the outflow from the right medial temporal pole to the amygdala were most important. Both for diagnosis and lateralization of the epilepsy connections in the theta and alpha band were most important.

## 4 Discussion

Using EEG-based directed functional connectivity and a RF classifier for automatic diagnosis and lateralization of TLE, we found that 1) both classifiers for diagnosis and lateralization of TLE achieved high accuracy (90%), 2) the outflows from the left and right hippocampus, and from the right ACC were the most important features for diagnosis and lateralization, respectively and 3) the interaction between features is important for a correct classification.

### 4.1 EEG-based connectivity measures for diagnosis and lateralization of TLE

This is the first study showing that functional connectivity using EEG without scalp pathological activity can be used for automatic diagnosis and lateralization of TLE. This could support TLE diagnosis in patients who do not show IEDs during routine scalp EEG recording. Furthermore, it could constitute a powerful lateralizing clinical aid in patients who are candidates for epilepsy surgery, especially in difficult cases where the currently used presurgical evaluation methods are not concordant.

We have built the classifier on directed functional connectivity measures and the comparison with other EEG network measures is beyond the scope of this study. Further comparative studies are warranted to assess the performance of classifiers based on other EEG metrics, such as undirected connectivity.

Likewise, future work will determine whether such classification system is efficient in patients with equivocal lateralisation or apparent bilateral TLE, who benefit from subsequent validation with invasive EEG.

Previous studies have used structural MRI, Diffusion Tensor Imaging (DTI), functional MRI (fMRI) or PET for automatic diagnosis and lateralization of TLE. In Table 4, we summarize the techniques, selected features and main findings of these studies. In TLE patients with Hippocampus Sclerosis (HS), Support Vector Machines (SVM) were applied to T1-weighted images and DTI (Focke et al., 2012). They achieved an accuracy of 100% for lateralization and an accuracy of 93% with a three-way SVM classifier (LTLE vs. RTLE vs. Controls). Excluding the contribution of the hippocampus yielded a lateralization accuracy of 92%, comparable to our results, but a lower diagnostic accuracy of 76%. It is noteworthy that this study solely included TLE patients with HS. However, HS is present only in 65% of surgical TLE population (Babb et al., 1984). In addition, patients with unilateral HS are, in general, less ambiguous cases. In our study, patients with other types of lesion or even patients with no detectable lesions were included, extending the use of our classifier to a more general population of TLE in which diagnosis can be more difficult. Unfortunately, due to the low number of patients without lesions in this study, a comparison between the classification accuracy in patients with epileptogenic lesions vs. patients without lesions could not be performed.

**Table 4:** Overview of automatic diagnosis of TLE in literature - comparison between several studies for automated diagnosis and lateralization of temporal lobe epilepsy.

	Subjects			Imaging	Features	Classifier	Diagnosis			Lateralization		
	LTLE	RTLE	Contr.				Acc. (%)	Sens. (%)	Spec. (%)	Acc. (%)	LTLE (%)	RTLE (%)
Focke et al., 2012	20	18	22	MRI	T1 and DTI image voxels with hippocampi masked out	SVM (lin)	93.2	89.5	100	100	100	100
							76.3	67.6	90.9	92.1	95.0	88.9
Kerr et al., 2013	39	34	32	Interictal PET	ROI metabolic changes	MLP	82.9	87.7	71.9	89.0	n/a	n/a
Kamiya et al., 2016	15	29	14	MRI	Network metrics from DTI structural connectomes	SVM (rbf)	n/a	n/a	n/a	86.4	89.7	80.0
Cantor-Rivera et al., 2015	9	8	19	MRI	ROI mean intensity and lateral asymmetry	SVM (lin)	88.9	82.4	94.7	n/a	n/a	n/a
Chiang et al., 2015	14	10	n/a	rs-fMRI	Functional connectivity network metrics	QDA	n/a	n/a	n/a	95.8	92.9	100
Yang et al., 2015	7	5	n/a	rs-fMRI	Functional connectivity values and network metrics	SVM (lin)	n/a	n/a	n/a	83.3	86.0	80.0
Current study	20	20	35	rs-EEG	Functional connectivity values>	RF	90.7	95.0	85.7	90.0	90.0	90.0

acc. = accuracy; sens. = sensitivity; spec. = specificity; n/a = not available; rs = resting state; lin = linear; rbf = radial basis function; QDA = quadratic discriminant analysis; MLP = multilayer perceptron.

In a FDG-PET study, interictal metabolic changes as input of the classifier (multilayer perceptron), led to an accuracy of 76% to simultaneously diagnose and lateralise TLE (Kerr et al., 2013). A SVM applied to graph theory measures obtained from DTI images, achieved an accuracy of 86.4% and an AUC of 0.91 for lateralization, but no results were reported for diagnosis (Kamiya et al., 2016). Using features from the T1-weighted MR images (Cantor-Rivera et al., 2015), a diagnostic accuracy of 88.9% was achieved with a linear SVM but no lateralization result was reported. Using resting-state fMRI and functional connectivity graph measures (Chiang et al., 2015), a lateralization classifier achieving 95.8% was built on a rather small set of subjects (14 LTLE and 10 RTLE patients). In another study, fMRI-based functional connectivity values and network metrics were used to lateralise TLE on a small cohort of patients (7 LTLE and 5 RLTE) (Yang et al., 2015). A linear SVM for lateralization gave an accuracy of 83.3%.

In this work, we obtained comparable or higher accuracies, sensitivities and specificities than those reported in these previous studies which used other imaging tools. Moreover, our results were obtained using 1 min of artefact-free EEG, extracted from a 10- to 15 min recording, which is less time-consuming than other imaging modalities. Due to the low cost and wide availability of EEG compared with other modalities, EEG-based measures could be widely implemented for diagnosis and lateralization. However, high-density EEG is not available in all epilepsy centers around the world, and therefore, future work should investigate whether this analysis would also yield a high accuracy when low-density EEG signals are used instead. In addition, our analysis was performed using eyes-closed resting-state recordings. Future work that includes eyes-opened resting state EEG recordings should elucidate whether our analysis would perform equally well during this condition.

In the study, we used a directed functional connectivity measure, wPDC, because 1) it allows us to depict the directionality of the connection, 2) it is a spectral measure, allowing to identify frequency specific features, 3) it depicts only direct interactions between brain regions, 4) it is a multivariate method, meaning that it considers all signals in the process simultaneously to compute the coefficients of the model, and 5) it has been shown to outperform PDC and enhance the physiological plausibility of the results (Plomp et al., 2014). However, the comparison between classification performance using wPDC and other connectivity measures would be interesting.

### 4.2 Main features for diagnosing and lateralising TLE

For the feature selection, although we preselected 14 regions based on our previous study (Coito et al., 2016), the selection of connections between these regions was done automatically, in a data-driven way and independently from prior clinical knowledge. This allowed us to identify new potential biomarkers for diagnosis and lateralization of TLE. We remark that the pre-selection of regions also has the disadvantage of missing potentially important regions for diagnosis and lateralization. The feature selection and classification system can be designed with randomly selected regions in order to search for potential biomarkers. This is however beyond the scope of the current study.

We showed that the outflow from the hippocampus and ACC were the best predictive features to automatically diagnose and lateralise TLE. This is concordant with our previous work on the connectivity pattern differences between LTLE, RTLE and healthy controls (Coito et al., 2016).

Indeed, the importance of the hippocampus and ACC in TLE has been widely recognized. The hippocampus has a pivotal role in the generation of interictal and ictal activity in the majority of TLE cases. Concordantly, many studies have reported reduced functional connectivity between both hippocampi, hippocampus and amygdala, or hippocampus and other regions of the Default-Mode Network, namely the ACC and the PCC (Coito et al., 2016, Pereira et al., 2010, Pittau et al., 2012, Zhang et al., 2010, Laufs et al., 2007, Liao et al., 2010). From a methodological perspective, there is converging evidence from intracranial and scalp EEG recordings that medial temporal lobe activity can be recorded with scalp EEG (Koessler et al., 2015, Nahum et al., 2011). A simultaneous scalp and intracranial study showed that purely medial temporal spikes could be detected on scalp recording (Koessler et al., 2015). Cognitive evoked potentials localised by icEEG in the hippocampus could also be localised in the medial temporal lobe using scalp evoked potential and electric source imaging (Nahum et al., 2011).

ACC functional connectivity has also been shown to be decreased in TLE patients compared to healthy controls (Coito et al., 2016, Stretton et al., 2015) and could be related to frequent mood disorders in TLE, since the ACC is a key node in the emotional processing network (Bush et al., 2000).

The classification allows us to identify features that are important to differentiate left vs right TLE patients and TLE vs controls. We found that especially connectivity in the theta and alpha band were important to diagnose and lateralize the epilepsy. The importance of the theta band is in correspondence with other studies. Douw et al. (2010) showed that theta band connectivity was altered in epilepsy patients and in brain tumor patients compared to controls. Future studies will have to show if the importance of the alpha leads to new biomarkers for diagnosis and lateralization of TLE.

### 4.3 Importance of feature interaction

Previous work used statistical tests to find features that had significantly different values in subjects with LTLE, RTLE and healthy subjects (Coito et al., 2016). However, these statistical analyses consider features individually, while the relevance of a connectivity value for classification depends also on which other connectivity values are considered as features.

The outflow from the right hippocampus to the left hippocampus and right amygdala were not significantly different for TLE compared with healthy controls, while they were among the most important features for classification. As shown in Fig. 1, these two connectivity values strongly interact with other features in the selection.

Although no significant differences in region-to-region directed functional connectivity were found between LTLE and RTLE, as also reported previously (Coito et al., 2016), the combination of these non-significant features seems to be sufficient for a good classification. Therefore, we show that classification algorithms that take into account the interaction between features can outperform significance tests between groups, which also allow us to find new biomarkers for diagnosis and lateralization of TLE.

## 5 Conclusion

The automatic diagnosis of TLE based on EEG periods without IEDs has several important advantages: (Laufs, 2012) resting-state EEG can be recorded in less than 1 h, overcoming long term monitoring and its related costs, (van Mierlo et al., 2013) no IEDs or ictal activity are required, enabling the use of this method in patients with low seizure and/or IEDs frequency, (Wilke et al., 2009) the features that result in the best classification provide insight into the differences between the groups (controls, LTLE and RTLE) and thus the mechanism of action of TLE.

The high accuracy achieved in this work for the automatic diagnosis of TLE based on functional connectivity measures using EEG periods without pathological activity shows that this approach could constitute a valuable bedside aid for clinicians. Our classification results were comparable to or better than earlier reported results using other imaging modalities. We showed that the outflows from the hippocampus and ACC are crucial features for the classifiers, in line with previous work showing the importance of these regions in TLE. The interaction between connectivity values are important for classification accuracy, even when connectivity values considered region by region might not be significantly different between groups. Further studies are warranted to determine whether our approach can help to lateralise epilepsy when bilateral epileptic activity is recorded.

## Appendix A Supplementary data

Supplementary material Image 1
