# Supplementary material for: Automated diagnosis of temporal lobe epilepsy in the absence of interictal spikes
Source: Neuroimage Clin. 2017 Sep 28;17:10–5. doi: 10.1016/j.nicl.2017.09.021 (PMC5842753; doi:10.1016/j.nicl.2017.09.021)
Supplement: Supplementary file 1 — Supplementary material [file mmc1.doc]

**Subjects, EEG recording and preprocessing**

Twenty LTLE patients, 20 RTLE patients and 35 healthy subjects were retrospectively selected from the high-density EEG database of the University Hospital of Geneva, University Hospital of Bern and Paracelsus Medical University in Salzburg according to the following inclusion criteria: drug-resistant TLE, unilateral anteromedial localization of the epileptogenic zone confirmed by good surgical outcome (Engel’s class I or II), intracranial EEG or concordant presurgical evaluation methods and the existence of at least a 10-15 min resting-state eyes-closed high-density EEG recording (96-256 channels). Twenty five patients had a 256-channel recording (12 in the LTLE group and 13 in the RTLE group), 10 patients had a 129-channel recording (6 in the LTLE group and 4 in the RTLE group) and 5 patients had a 96-channel recording (2 in the LTLE group and 3 in the RTLE group).

The clinical details of the patients can be found in the Table A.

Healthy subjects underwent a resting-state eyes-closed recording using an EEG system (*Electrical Geodesics* system) with 256 electrodes. In both healthy subjects and patient who had a 256-channel recording, we removed the facial electrodes as well as the electrodes on the neck since those usually contain artefacts from facial muscles movements and lower impedances, ending up therefore with the signals from 204 electrodes for analysis.

All datasets were filtered offline between 1 and 100 Hz and then downsampled to 250 Hz. We visually inspected all analysed signals and topographies for bad channels, which were interpolated using the 3D splines method, as implemented in the freely available software Cartool (http://brainmapping.unige.ch/cartool). Sixty epochs of 1 second, free of artefacts and IEDs, during wakefulness were selected per subject.

Table A – Patient’s clinical details (P1-P20 LTLE patients; P21-P40 RTLE patients).

| **Patient** | **Gender** | **Age (y)** | **Age of epilepsy onset (y)** | **Focus Side** | **Structural epileptogenic lesion on MRI** | **Surgery Outcome (Engel’s class)** | **Intracranial EEG** |
| --- | --- | --- | --- | --- | --- | --- | --- |
| P1 | F | 15 | 9 | Left | Tumor | I | No |
| P2 | M | 33 | 15 | Left | Normal | II | Yes |
| P3 | F | 47 | 3 | Left | HS | I | No |
| P4 | F | 15 | 9 months | Left | HS | I | Yes |
| P5 | F | 41 | 3 | Left | HS | n.a. | No |
| P6 | F | 25 | 20 | Left | Normal | n.a. | No |
| P7 | M | 18 | 13 | Left | Normal | I | Yes |
| P8 | M | 35 | 27 | Left | HS | I | No |
| P9 | M | 16 | 1 | Left | Amyg-Hipp dysplasia | I | No |
| P10 | M | 15 | 11 | Left | HS + left TA | I | No |
| P11 | F | 15 | 3 | Left | Left TA | I | No |
| P12 | F | 40 | 12 | Left | HS | I | No |
| P13 | F | 20 | 5 | Left | HS | I | No |
| P14 | M | 31 | 29 | Left | Normal | I | Yes |
| P15 | M | 45 | 17 | Left | HS | I | No |
| P16 | F | 29 | 26 | Left | Amyg-Hipp DNET | I | No |
| P17 | M | 53 | 2 | Left | HS | I | No |
| P18 | F | 37 | 28 | Left | Normal | I | No |
| P19 | F | 25 | 20 | Left | Cortical dysplasia | n.a. | Yes |
| P20 | M | 18 | 8 | Left | HS | I | No |
| P21 | F | 27 | 27 | Right | Amyg DNET | I | No |
| P22 | F | 53 | 25 | Right | Hypertrophy right Amygdala | n.a. | No |
| P23 | F | 36 | 6 months | Right | HS | I | No |
| P24 | M | 37 | 20 | Right | HS | I | No |
| P25 | F | 50 | 31 | Right | HS + right TA | II | No |
| P26 | F | 44 | 20 | Right | HS | II | No |
| P27 | M | 34 | 7 | Right | HS | II | No |
| P28 | F | 55 | 4 | Right | HS | I | No |
| P29 | M | 35 | 22 | Right | Normal | n.a. | Yes |
| P30 | M | 18 | 12 | Right | HS | I | No |
| P31 | F | 37 | 25 | Right | HS | I | No |
| P32 | M | 16 | 11 | Right | HS | I | Yes |
| P33 | F | 30 | 25 | Right | Dysplasia amygdala | I | No |
| P34 | M | 27 | 5 | Right | HS | I | No |
| P35 | M | 27 | 18 | Right | Dysplasia hippocampus + gyrus lingualis | I | Yes |
| P36 | F | 57 | 15 | Right | HS | I | Yes |
| P37 | M | 24 | 17 | Right | HS | I | No |
| P38 | F | 30 | 27 | Right | HS | n.a. | No |
| P39 | M | 50 | 11 | Right | HS | I | Yes |
| P40 | M | 16 | 11 | Right | Amygdala Dysplasia | II | No |

F - Female; M- Men; HS - hippocampus sclerosis; TA - temporal atrophy

**Electrical Source Imaging and Selection of Regions of Interest**

The activity of brain sources during the selected EEG epochs was obtained using Electrical Source Imaging (ESI). The forward model was constructed based on a simplified realistic head model using each individual’s T1-weighted MRI with consideration of skull thickness (Locally Spherical Model with Anatomical Constraints, LSMAC ). Around 5000 solution points were equally distributed in the grey matter. A linear distributed inverse solution with biophysical constraints was used to calculate the 3D current source density (Local Auto-Regressive Averages, LAURA [^3^](#_ENREF_3).

The grey matter was parcelled in 82 Regions Of Interest (ROI) based on the automated anatomical labelling digital atlas [^4^](#_ENREF_4) after normalization to the MNI space using SPM8 (www.fil.ion.ucl.ac.uk/spm). The solution point closest to the centroid of each ROI was considered as representative of the source activity in this ROI to reduce the dimensionality of the solution space. In order to take the time-varying three-dimensional orientation of the source dipoles into account, as well as to obtain a scalar time-series from the 3D dipole time-series, these were projected onto the predominant dipole direction of each ROI over all epochs . This procedure resulted in 82 time-series representing the activity of each individual ROI during the 60 selected epochs.

**Directed Functional Connectivity**

Directed functional connectivity is commonly assessed using the concept of Granger-causality: a signal is said to Granger-cause another if the knowledge of the past of the former reduces the prediction error of the present of the latter [^7^](#_ENREF_7). One of the multivariate approaches to estimate brain connectivity in the frequency domain using the concept of Granger-causality is Partial Directed Coherence (PDC) [^8^](#_ENREF_8). PDC estimates the directional and direct interactions between all signals in a multivariate process. It is computed using multivariate autoregressive MVAR models of an appropriate order, which simultaneously models multiple time-series, here the source signals obtained in the 82 ROIs. We used a model order of 10, corresponding to 40 ms of the signal, in concordance with previous studies with similar epoch length and sampling frequency . The MVAR model coefficients were computed using the Nutall-Strand algorithm . We computed the squared PDC [^13^](#_ENREF_13) normalized with respect to the inflows [^14^](#_ENREF_14) and then scaled the results by weighting with the normalized spectral power of the source region (weighted PDC, wPDC [^14^](#_ENREF_14)). To obtain the spectral power we computed the Fast Fourier Transform (FFT) for each electrode, applied ESI to the real and imaginary part of the FFT separately and then combined them to avoid frequency doubling . The mean spectral power was obtained for each patient and scaled (0-1, in the same way as PDC) across ROIs and frequencies (1-40 Hz). In this way, we used the spectral power of the signal to weight the connectivity matrices [^14^](#_ENREF_14).

Given the 20mm spatial accuracy of ESI for localizing interictal epileptic activity [^17^](#_ENREF_17), the outflows seen in the amygdala, hippocampus and parahippocampal gyrus should not be considered strictly independent but rather globally as medial temporal lobe activity.

**Feature Selection and Classification with AUC and Balanced Random Forests**

In contrast to the original feature selection method by Genuer et al. [^18^](#_ENREF_18), we used the area under the receiver operating characteristic curve (AUC) instead of classification accuracy for evaluation of the binary classifiers. The curve plots the true positive rate (sensitivity) against the false positive rate (1-specificity) of the classifier at various classification thresholds and thus uses the assigned probability that a subject belongs to a certain class rather than the assigned class itself. Therefore it is known to be a more complete evaluation metric for binary classifiers compared to the classification accuracy [^19^](#_ENREF_19).

The advantage of using RF as classification technique for the automatic diagnosis of diseases is multifold. First, it is known to manage classification problems with a low number of recorded input samples in contrast to a high number of feature values per input. As such it has been shown to outperform other classification techniques, such as SVMs and Logistic Regression . Secondly, the samples that do not appear in the bootstrap subset (called ‘out-of-bag samples’) can be used to test the trees on unseen inputs without the need for extra samples in a separate test set. In automatic diagnosis this avoids the high cost of recording extra subjects for testing the system. Finally, the decision trees and their aggregation by voting make the internal classification mechanism transparent and easy to understand, which is important for integration of automatic diagnosis systems in clinical practice.

RF also lends itself as an ideal technique for the selection of features. The performance of the forest on the out-of-bag subjects can be used to assign an importance value to each feature incorporating the interaction between features. Importance values were used for feature selection and interpretation.

A downside of RF is that its performance is known to suffer from class-imbalance in the dataset, as is the case for our diagnosis where we have 40 TLE patients compared to 35 healthy controls. We tried to compensate for this limitation by using a slightly adapted version of RF: Balanced Random Forests, where balanced bootstrap subsets were used to train every tree in the ensemble. Every forest contained 1000 trees. The size of the random set of features from which splits were chosen was log_2_(M), where M is the total amount of features per subject.

**Evaluation of the classifier**

The confusion matrix in Table II should be interpreted with care as it gives three-class classification results while no three-class classifier was used in this work. One could for example interpret the last row of the matrix as an indicator that the RTLE and controls are close to each other and then find it remarkable that no RTLE subjects were misclassified as control. It is for example known that resting-state EEG is based more on right hemisphere structures [^22^](#_ENREF_22). However, following the two-step procedure in this work, we conclude that these five control subjects were close to the TLE class and that the lateralization classifier seems to be biased towards the RTLE class for these misclassified control subjects. A possible reason for this bias is the high occurrence of right hemisphere regions in the selected features for lateralization. We could take this into account during feature selection for lateralization but expect the lateralization performance on true TLE subjects to suffer from that.

We have chosen to evaluate the classifier in a LOOCV procedure as this closely resembles its reliability in a real clinical setting. In this setting, the classifier will be updated following the inclusion of a new subject and the newly determined parameters of the system will be applied to the next test subject. In this way the maximum amount of available knowledge is used to design the classifier. LOOCV replicates this procedure for different sets of available data and different test subjects. The results then show on average how well the classifier can generalize the knowledge from the existing database of subjects to a new unseen subject.

**References**

1. Brunet D, Murray MM, Michel CM. Spatiotemporal analysis of multichannel EEG: CARTOOL. Comput Intell Neurosci 2011;2011:813870.

2. Birot G, Spinelli L, Vulliemoz S, et al. Head model and electrical source imaging: a study of 38 epileptic patients. NeuroImage Clinical 2014;5:77-83.

3. Grave de Peralta Menendez R, Murray MM, Michel CM, Martuzzi R, Gonzalez Andino SL. Electrical neuroimaging based on biophysical constraints. NeuroImage 2004;21:527-539.

4. Tzourio-Mazoyer N, Landeau B, Papathanassiou D, et al. Automated anatomical labeling of activations in SPM using a macroscopic anatomical parcellation of the MNI MRI single-subject brain. NeuroImage 2002;15:273-289.

5. Coito A, Plomp G, Genetti M, et al. Dynamic directed interictal connectivity in left and right temporal lobe epilepsy. Epilepsia 2015;56:207-217.

6. Plomp G, Hervais-Adelman A, Astolfi L, Michel CM. Early recurrence and ongoing parietal driving during elementary visual processing. Sci Rep 2015;5:18733.

7. Granger CWJ. Investigating Causal Relations by Econometric Models and Cross-spectral Methods. Econometrica 1969;37:424-438.

8. Baccala LA, Sameshima K. Partial directed coherence: a new concept in neural structure determination. Biol Cybern 2001;84:463-474.

9. Astolfi L, Cincotti F, Mattia D, et al. Tracking the time-varying cortical connectivity patterns by adaptive multivariate estimators. IEEE transactions on bio-medical engineering 2008;55:902-913.

10. Coito A, Genetti M, Pittau F, et al. Altered directed functional connectivity in temporal lobe epilepsy in the absence of interictal spikes: A high density EEG study. Epilepsia 2016;57:402-411.

11. Marple SL. Digital Spectral Analysis with Applications: Prentice-Hall, Englewood Cliffs, NJ, 1987.

12. Schlögl A. A comparison of multivariate autoregressive estimators. Signal Processing 2006;86:2426-2429.

13. Astolfi L, Cincotti F, Mattia D, et al. Assessing cortical functional connectivity by partial directed coherence: simulations and application to real data. IEEE transactions on bio-medical engineering 2006;53:1802-1812.

14. Plomp G, Quairiaux C, Michel CM, Astolfi L. The physiological plausibility of time-varying Granger-causal modeling: Normalization and weighting by spectral power. NeuroImage 2014.

15. Koenig T P-MR. Multichannel frequency and time-frequency analysis. In: Michel CMK, T.; Brandeis, D.; Gianotti, L.R.R.; Wackermann, J., ed. Electrical Neuroimaging: Cambridge Medicine, 2009.

16. Frei E, Gamma A, Pascual-Marqui R, Lehmann D, Hell D, Vollenweider FX. Localization of MDMA-induced brain activity in healthy volunteers using low resolution brain electromagnetic tomography (LORETA). Human brain mapping 2001;14:152-165.

17. Megevand P, Spinelli L, Genetti M, et al. Electric source imaging of interictal activity accurately localises the seizure onset zone. Journal of neurology, neurosurgery, and psychiatry 2014;85:38-43.

18. Genuer R, Poggi JM, Tuleau-Malot C. Variable selection using random forests. Pattern Recognition Letters 2010;31:2225-2236.

19. Huang J, Ling CX. Using AUC and accuracy in evaluating learning algorithms. IEEE Transactions on Knowledge and Data Engineering 2005;17:299-310.

20. Khalilia M, Chakraborty S, Popescu M. Predicting disease risks from highly imbalanced data using random forest. BMC medical informatics and decision making 2011;11:51.

21. Ozcift A, Gulten A. Classifier ensemble construction with rotation forest to improve medical diagnosis performance of machine learning algorithms. Computer methods and programs in biomedicine 2011;104:443-451.

22. Chiron C, Jambaque I, Nabbout R, Lounes R, Syrota A, Dulac O. The right brain hemisphere is dominant in human infants. Brain : a journal of neurology 1997;120 ( Pt 6):1057-1065.
